# Crystal structure of tris­(di­methyl­amido-κ*N*)­bis­(di­methyl­amine-κ*N*)­zirconium(IV) iodide

**DOI:** 10.1107/S2056989015023919

**Published:** 2016-01-01

**Authors:** Wesley D. Clark, Gopalakrishna Akurathi, Henry U. Valle, T. Keith Hollis

**Affiliations:** a Department of Chemistry, Mississippi State University, Mississippi State, 39762, USA

**Keywords:** crystal structure, zirconium, amido ligands, iodide, di­methyl­amine, N—H⋯I inter­actions

## Abstract

Zirconium amides have become increasingly popular and useful due to their widespread use as precursors to other zirconium complexes and their use in the production of solid oxide fuel cells (SOFCs). Herein we report the mol­ecular structure of tris­(di­methyl­amido)­bis­(di­methyl­amine)­zirconium(IV) iodide, [Zr(C_2_H_6_N)_3_(C_2_H_7_N)_2_]I. The bond lengths and bond angles are consistent with a slightly distorted trigonal–bipyramidal coordination geometry around the metal atom. N⋯I contacts of 3.6153 (15) and 3.5922 (14) Å are consistent with the presence of N—H⋯I inter­actions. These N—H⋯I inter­actions link the complex cations and iodide anions into extended chains that propagate parallel to the *a* axis.

## Abstract 2

The crystal structure of tris­(di­methyl­amido)­bis­(di­methyl­amine) zirconium(IV) iodide is reported.

## Chemical context   

Zirconium amide complexes are widely used in the synthesis of other zirconium complexes and solid oxide fuel cells (SOFCs). Additionally, many zirconium amide complexes are precatalysts for hydro­amination/cyclization of unactivated amino­alkenes (Luconi *et al.*, 2013 ▸, Manna *et al.*, 2013 ▸ and references therein). Perhaps one of the most well known zirconium amide complexes is tetra­kis­(di­methyl­amido)­zirconium(IV). The title compound serendipitously formed from the reaction of an excess of tetra­kis­(di­methyl­amido)­zirconium(IV) and a bis­(imidazo­l­ium) salt that we routinely perform, as illustrated in the Scheme below.
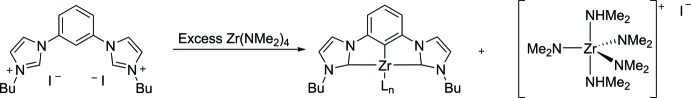


## Structural commentary   

**Figure 1:**
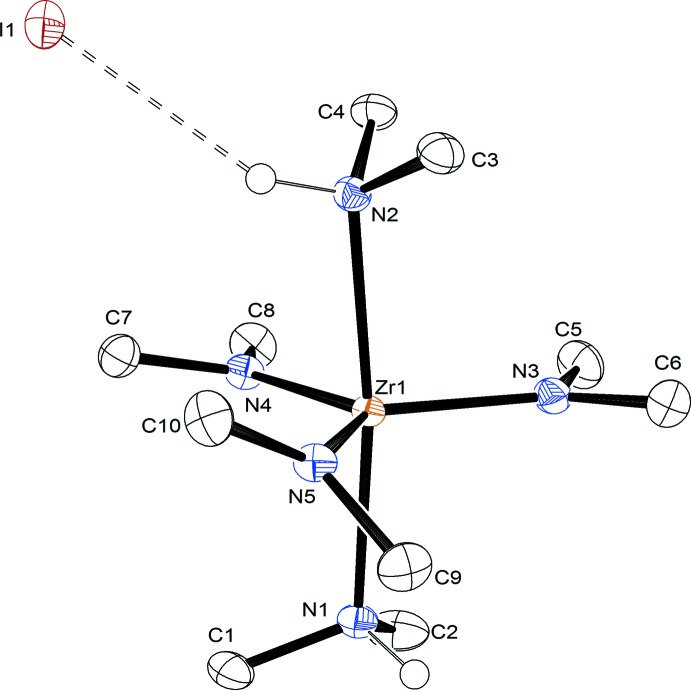
Displacement ellipsoid plot of the title compound. All hydrogens except the amine H atoms have been omitted for clarity. Ellipsoids are shown at the 50% probability level.

The zirconium complex has a slightly distorted trigonal–bipyramidal geometry with three dimethamido ligands in equatorial positions and two dimethyamine ligands in axial positions (Fig. 1 ▸). Iodide provides a counterbalancing charge for the cationic zirconium complex. The Zr—amine bonds [Zr1—N1 and Zr1—N2, 2.3730 (13) and 2.3695 (14) Å, respectively] are significantly longer than those of the amide ligands [Zr1—N3 2.0249 (14), Zr1—N4 2.0393 (14), and Zr1—N5 2.0389 (14) Å]. The C—N bonds vary little, with the shortest and longest bond being only 0.026 (2) Å different [N1–C2 1.480 (2) and N3—C5 1.454 (2) Å]. The N1—Zr1—N2 angle of 172.83 (5)° and the N1—Zr1—N3 of 94.35 (5)° deviate slightly from the ideal angles of trigonal–bipyramidal geometry. The N3—Zr1—N5, N3—Zr1—N4, and N4—Zr1—N5 angles are close to 120° [116.76 (6), 120.99 (6), and 122.15 (6)°, respectively]. The C—N—Zr angles vary with the smallest and largest angles being almost 20° different [C10—N5—Zr1 135.34 (11) and C1—N1—Zr1 110.52 (10)°]. The amine nitro­gen atoms (N1 and N2) are puckered in the structure [Zr1—N1—C1—C2 −124.71 (15) and Zr1—N2—C3—C4 127.27 (15)°]. This is in contrast to the amide ligands which are essentially coplanar with the metal [Zr1—N3—C5—C6 175.88 (19), Zr1—N4—C7—C8 174.05 (17), and Zr1—N5—C9—C10 −176.79 (17)°]. One amide ligand is twisted out of the plane by roughly 40° [C9—N5—Zr1—N3 −39.10 (13)°].

## Supra­molecular features   

**Figure 2:**
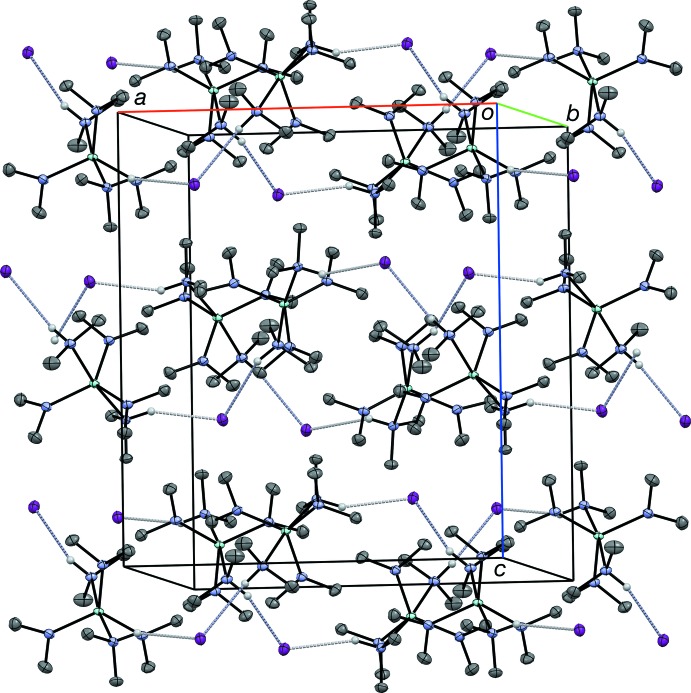
A packing plot of the unit cell viewed approximately down the *b* axis, illustrating the N—H⋯I inter­actions (grey dotted lines). All hydrogen atoms except the amine H atoms have been omitted for clarity. Displacement ellipsoids are shown at the 50% probability level.

N⋯I contacts of 3.6153 (15) and 3.5922 (14) Å are consistent with the presence of N—H⋯I inter­actions (Table 1 ▸). The ‘twist’ of the second di­methyl­amido ligand away from the first is consistent with inter­action with a symmetry-related I^−^ atom (H2—N2—N1—H1 − 114°; Fig. 2 ▸). The N—H⋯I inter­actions link the complex cations and iodide anions into extended chains that propagate parallel to the *a* axis.

## Database survey   

The synthesis or crystal structure of tris­(di­methyl­amido)­bis(di­methyl­amine)­zirconium(IV) iodide has not been reported as of 22 April 2015 based on a comprehensive WebCSD and Scifinder Scholar search. Similar compounds have been characterized crystallographically, for example tetra­kis­(di­methyl­amido)­zirconium(IV) and its lithium di­methyl­amido adduct (Chisholm *et al.*, 1988 ▸) and several more zirconium-amide iodide complexes (Lehn & Hoffman, 2002 ▸).

## Synthesis and crystallization   

1,3-Bis(3′-hexyl­imidazol-1′-yl)benzene diiodide (301 mg, 0.475 mmol), tetra­kis­(di­methyl­amido)­zirconium(IV) (317 mg, 1.24 mmol) and dry toluene (2.8 mL) were combined in an inert atmosphere of Ar and heated at 383 K for 5 min in a sealed screw-cap vial. While heating, the reaction mixture became homogeneous. Upon cooling to room temperature, an oil formed. The top layer was removed and the oil was washed with toluene (3 × 3 mL). The toluene washings were combined and allowed to sit at room temperature. Colorless crystals formed after 2 months. The mother liquor was deca­nted and the crystals were covered with paratone oil after using a few crystals for ^1^H NMR spectroscopy. ^1^H NMR spectra of the samples indicated that 2-[1,3-bis­(3′-hexyl-imidazol-2′-yl­idene)phenyl­ene](di­methyl­amido)­diiodidozirconium(IV) and 2-[1,3-bis­(3′-hexyl-imidazol-2′-yl­idene)phenyl­ene]bis­(di­methyl­amido)iodidozirconium(IV) had crystallized in the form of needles, which were not suitable for single-crystal X-ray diffraction. However, a suitable tablet-shaped crystal of tris­(di­methyl­amido)­bis­(di­methyl­ammine)zirconium(IV) iodide was selected, mounted, and analyzed.

## Refinement   

Crystal data, data collection and structure refinement details are summarized in Table 2 ▸. H atoms bonded to C and N atoms were placed at geometrically calculated positions and refined using a riding model: C—H = 0.98, N—H = 1.00 Å; *U*_iso_(H) = 1.5*U*_eq_(C) or 1.2*U*_eq_(N).

## Supplementary Material

Crystal structure: contains datablock(s) I. DOI: 10.1107/S2056989015023919/pk2563sup1.cif

Structure factors: contains datablock(s) I. DOI: 10.1107/S2056989015023919/pk2563Isup2.hkl

CCDC reference: 1037746

Additional supporting information:  crystallographic information; 3D view; checkCIF report

## Figures and Tables

**Table 1:** Hydrogen-bond geometry (Å, °)

*D*—H⋯*A*	*D*—H	H⋯*A*	*D*⋯*A*	*D*—H⋯*A*
N1—H1⋯I1^i^	1.00	2.78	3.5922 (14)	138
N2—H2⋯I1	1.00	2.69	3.6153 (15)	138

Symmetry code: (i) 
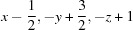
.

**Table 2:** Experimental details

Crystal data	
Chemical formula	[Zr(C_2_H_7_N)_2_(C_2_H_6_N)_3_]I
*M*_r_	440.52
Crystal system, space group	Orthorhombic, *P* *b* *c* *a*
Temperature (K)	100
*a*, *b*, *c* (Å)	14.2425 (3), 15.4113 (3), 16.8537 (3)
*V* (Å^3^)	3699.31 (12)
*Z*	8
Radiation type	Mo *K*α
μ (mm^−1^)	2.26
Crystal size (mm)	0.2 × 0.1 × 0.1

Data collection	
Diffractometer	Bruker APEXII CCD
Absorption correction	Numerical (*SADABS*; Bruker, 2014 ▸)
*T*_min_, *T*_max_	0.656, 0.745
No. of measured, independent and observed [*I* > 2σ(*I*)] reflections	29665, 3620, 3319
*R*_int_	0.027
(sin θ/λ)_max_ (Å^−1^)	0.617

Refinement	
*R*[*F* ^2^ > 2σ(*F* ^2^)], *wR*(*F* ^2^), *S*	0.015, 0.036, 1.07
No. of reflections	3620
No. of parameters	154
H-atom treatment	H-atom parameters constrained
Δρ_max_, Δρ_min_ (e Å^−3^)	0.33, −0.34

Computer programs: *APEX2* and *SAINT* (Bruker, 2013 ▸), *SHELXS* and *SHELXL* (Sheldrick, 2008 ▸), *OLEX2* (Dolomanov *et al.*, 2009 ▸), *Mercury* (Macrae *et al.*, 2006 ▸) and *publCIF* (Westrip, 2010 ▸).

## supplementary crystallographic information

### Crystal data

**Table:**

[Zr(C_2_H_6_N)_3_(C_2_H_7_N)_2_]I	*D*_x_ = 1.582 Mg m^−^^3^
*M**_r_* = 440.52	Mo *K*α radiation, λ = 0.71073 Å
Orthorhombic, *P**b**c**a*	Cell parameters from 9970 reflections
*a* = 14.2425 (3) Å	θ = 2.3–26.0°
*b* = 15.4113 (3) Å	µ = 2.26 mm^−^^1^
*c* = 16.8537 (3) Å	*T* = 100 K
*V* = 3699.31 (12) Å^3^	Tablet, colourless
*Z* = 8	0.2 × 0.1 × 0.1 mm
*F*(000) = 1760	

### Data collection

**Table:**

Bruker APEXII CCD diffractometer	3319 reflections with *I* > 2σ(*I*)
φ and ω scans	*R*_int_ = 0.027
Absorption correction: numerical (*SADABS*; Bruker, 2014)	θ_max_ = 26.0°, θ_min_ = 2.3°
*T*_min_ = 0.656, *T*_max_ = 0.745	*h* = −17→17
29665 measured reflections	*k* = −18→19
3620 independent reflections	*l* = −18→20

### Refinement

**Table:**

Refinement on *F*^2^	0 restraints
Least-squares matrix: full	Hydrogen site location: inferred from neighbouring sites
*R*[*F*^2^ > 2σ(*F*^2^)] = 0.015	H-atom parameters constrained
*wR*(*F*^2^) = 0.036	*w* = 1/[σ^2^(*F*_o_^2^) + (0.0158*P*)^2^ + 1.6295*P*] where *P* = (*F*_o_^2^ + 2*F*_c_^2^)/3
*S* = 1.07	(Δ/σ)_max_ = 0.002
3620 reflections	Δρ_max_ = 0.33 e Å^−^^3^
154 parameters	Δρ_min_ = −0.34 e Å^−^^3^

### Special details

**Table:**

Experimental. wR2(int) was 0.0590 before and 0.0411 after absorption correction. The ratio of minimum to maximum transmission is 0.8806. The λ/2 correction factor is 0.00150.
Geometry. All e.s.d.'s (except the e.s.d. in the dihedral angle between two l.s. planes) are estimated using the full covariance matrix. The cell e.s.d.'s are taken into account individually in the estimation of e.s.d.'s in distances, angles and torsion angles; correlations between e.s.d.'s in cell parameters are only used when they are defined by crystal symmetry. An approximate (isotropic) treatment of cell e.s.d.'s is used for estimating e.s.d.'s involving l.s. planes.

### Fractional atomic coordinates and isotropic or equivalent isotropic displacement parameters (Å^2^)

**Table:**

	*x*	*y*	*z*	*U*_iso_*/*U*_eq_	
C1	0.42535 (13)	0.59533 (12)	0.45204 (11)	0.0250 (4)	
H1A	0.4484	0.6453	0.4219	0.037*	
H1B	0.4754	0.5737	0.4867	0.037*	
H1C	0.4062	0.5494	0.4153	0.037*	
C2	0.30833 (15)	0.54710 (12)	0.54677 (12)	0.0314 (5)	
H2A	0.2545	0.5655	0.5788	0.047*	
H2B	0.2888	0.5010	0.5103	0.047*	
H2C	0.3580	0.5253	0.5817	0.047*	
C3	0.39168 (13)	0.94138 (11)	0.64365 (11)	0.0208 (4)	
H3A	0.3892	0.9531	0.5865	0.031*	
H3B	0.3277	0.9361	0.6644	0.031*	
H3C	0.4240	0.9892	0.6706	0.031*	
C4	0.44722 (13)	0.84138 (11)	0.74390 (10)	0.0210 (4)	
H4A	0.4814	0.7871	0.7531	0.031*	
H4B	0.4796	0.8891	0.7709	0.031*	
H4C	0.3833	0.8360	0.7648	0.031*	
C5	0.23350 (14)	0.69989 (13)	0.70093 (11)	0.0279 (4)	
H5A	0.2871	0.6622	0.7130	0.042*	
H5B	0.2214	0.7383	0.7461	0.042*	
H5C	0.1779	0.6642	0.6908	0.042*	
C6	0.17635 (12)	0.80851 (13)	0.61133 (12)	0.0260 (4)	
H6A	0.1921	0.8427	0.5642	0.039*	
H6B	0.1204	0.7735	0.6006	0.039*	
H6C	0.1639	0.8476	0.6560	0.039*	
C7	0.57938 (12)	0.68391 (12)	0.60513 (11)	0.0222 (4)	
H7A	0.5755	0.7251	0.5609	0.033*	
H7B	0.6177	0.7089	0.6477	0.033*	
H7C	0.6081	0.6298	0.5867	0.033*	
C8	0.48782 (14)	0.60495 (12)	0.70102 (11)	0.0249 (4)	
H8A	0.4239	0.5941	0.7200	0.037*	
H8B	0.5160	0.5503	0.6832	0.037*	
H8C	0.5256	0.6294	0.7442	0.037*	
C9	0.33176 (13)	0.81902 (12)	0.42983 (11)	0.0235 (4)	
H9A	0.2804	0.7826	0.4495	0.035*	
H9B	0.3103	0.8793	0.4258	0.035*	
H9C	0.3515	0.7984	0.3774	0.035*	
C10	0.48868 (13)	0.86737 (11)	0.45761 (11)	0.0233 (4)	
H10A	0.5408	0.8630	0.4954	0.035*	
H10B	0.5094	0.8471	0.4054	0.035*	
H10C	0.4683	0.9279	0.4537	0.035*	
N1	0.34417 (10)	0.62186 (9)	0.50068 (8)	0.0166 (3)	
H1	0.2932	0.6412	0.4639	0.020*	
N2	0.44334 (10)	0.85934 (9)	0.65782 (8)	0.0153 (3)	
H2	0.5094	0.8692	0.6399	0.018*	
N3	0.25455 (10)	0.75170 (9)	0.63104 (8)	0.0180 (3)	
N4	0.48491 (10)	0.66602 (9)	0.63514 (8)	0.0162 (3)	
N5	0.41111 (10)	0.81422 (9)	0.48479 (8)	0.0159 (3)	
Zr1	0.38438 (2)	0.74109 (2)	0.58304 (2)	0.01200 (5)	
I1	0.68609 (2)	0.92790 (2)	0.65905 (2)	0.02035 (4)	

### Atomic displacement parameters (Å^2^)

**Table:**

	*U*^11^	*U*^22^	*U*^33^	*U*^12^	*U*^13^	*U*^23^
C1	0.0281 (10)	0.0224 (10)	0.0244 (10)	0.0032 (8)	−0.0005 (8)	−0.0092 (8)
C2	0.0509 (14)	0.0178 (9)	0.0256 (11)	−0.0139 (9)	−0.0001 (9)	0.0003 (8)
C3	0.0240 (10)	0.0157 (8)	0.0228 (10)	0.0010 (7)	−0.0006 (8)	−0.0027 (7)
C4	0.0258 (10)	0.0226 (9)	0.0146 (9)	−0.0021 (7)	−0.0008 (7)	−0.0024 (7)
C5	0.0300 (11)	0.0307 (10)	0.0231 (10)	−0.0020 (8)	0.0079 (8)	0.0020 (8)
C6	0.0211 (10)	0.0283 (10)	0.0287 (11)	−0.0001 (8)	−0.0007 (8)	−0.0021 (8)
C7	0.0212 (10)	0.0229 (9)	0.0224 (10)	0.0000 (7)	−0.0011 (8)	0.0017 (7)
C8	0.0322 (11)	0.0215 (9)	0.0211 (10)	0.0050 (8)	−0.0011 (8)	0.0070 (8)
C9	0.0279 (10)	0.0207 (9)	0.0220 (10)	0.0016 (8)	−0.0057 (8)	0.0043 (7)
C10	0.0255 (10)	0.0208 (9)	0.0236 (10)	−0.0017 (7)	0.0052 (8)	0.0063 (7)
N1	0.0209 (8)	0.0144 (7)	0.0145 (7)	−0.0017 (6)	−0.0003 (6)	−0.0003 (6)
N2	0.0166 (7)	0.0154 (7)	0.0138 (7)	−0.0006 (6)	0.0009 (6)	−0.0006 (5)
N3	0.0177 (8)	0.0205 (8)	0.0157 (7)	−0.0025 (6)	0.0025 (6)	−0.0022 (6)
N4	0.0202 (8)	0.0147 (7)	0.0136 (7)	0.0011 (6)	−0.0014 (6)	0.0021 (6)
N5	0.0195 (8)	0.0137 (7)	0.0145 (7)	−0.0018 (6)	−0.0005 (6)	0.0013 (5)
Zr1	0.01412 (9)	0.01104 (8)	0.01085 (9)	−0.00094 (6)	0.00062 (6)	0.00062 (6)
I1	0.01689 (7)	0.02028 (7)	0.02389 (8)	0.00079 (4)	0.00159 (4)	0.00231 (4)

### Geometric parameters (Å, º)

**Table:**

C1—H1A	0.9800	C7—H7A	0.9800
C1—H1B	0.9800	C7—H7B	0.9800
C1—H1C	0.9800	C7—H7C	0.9800
C1—N1	1.475 (2)	C7—N4	1.464 (2)
C2—H2A	0.9800	C8—H8A	0.9800
C2—H2B	0.9800	C8—H8B	0.9800
C2—H2C	0.9800	C8—H8C	0.9800
C2—N1	1.480 (2)	C8—N4	1.456 (2)
C3—H3A	0.9800	C9—H9A	0.9800
C3—H3B	0.9800	C9—H9B	0.9800
C3—H3C	0.9800	C9—H9C	0.9800
C3—N2	1.482 (2)	C9—N5	1.463 (2)
C4—H4A	0.9800	C10—H10A	0.9800
C4—H4B	0.9800	C10—H10B	0.9800
C4—H4C	0.9800	C10—H10C	0.9800
C4—N2	1.478 (2)	C10—N5	1.450 (2)
C5—H5A	0.9800	N1—H1	1.0000
C5—H5B	0.9800	N1—Zr1	2.3730 (13)
C5—H5C	0.9800	N2—H2	1.0000
C5—N3	1.454 (2)	N2—Zr1	2.3695 (14)
C6—H6A	0.9800	N3—Zr1	2.0249 (14)
C6—H6B	0.9800	N4—Zr1	2.0393 (14)
C6—H6C	0.9800	N5—Zr1	2.0389 (14)
C6—N3	1.455 (2)		
			
H1A—C1—H1B	109.5	N4—C8—H8B	109.5
H1A—C1—H1C	109.5	N4—C8—H8C	109.5
H1B—C1—H1C	109.5	H9A—C9—H9B	109.5
N1—C1—H1A	109.5	H9A—C9—H9C	109.5
N1—C1—H1B	109.5	H9B—C9—H9C	109.5
N1—C1—H1C	109.5	N5—C9—H9A	109.5
H2A—C2—H2B	109.5	N5—C9—H9B	109.5
H2A—C2—H2C	109.5	N5—C9—H9C	109.5
H2B—C2—H2C	109.5	H10A—C10—H10B	109.5
N1—C2—H2A	109.5	H10A—C10—H10C	109.5
N1—C2—H2B	109.5	H10B—C10—H10C	109.5
N1—C2—H2C	109.5	N5—C10—H10A	109.5
H3A—C3—H3B	109.5	N5—C10—H10B	109.5
H3A—C3—H3C	109.5	N5—C10—H10C	109.5
H3B—C3—H3C	109.5	C1—N1—C2	110.24 (14)
N2—C3—H3A	109.5	C1—N1—H1	107.9
N2—C3—H3B	109.5	C1—N1—Zr1	110.52 (10)
N2—C3—H3C	109.5	C2—N1—H1	107.9
H4A—C4—H4B	109.5	C2—N1—Zr1	112.27 (11)
H4A—C4—H4C	109.5	Zr1—N1—H1	107.9
H4B—C4—H4C	109.5	C3—N2—H2	106.8
N2—C4—H4A	109.5	C3—N2—Zr1	113.23 (10)
N2—C4—H4B	109.5	C4—N2—C3	109.66 (13)
N2—C4—H4C	109.5	C4—N2—H2	106.8
H5A—C5—H5B	109.5	C4—N2—Zr1	113.04 (10)
H5A—C5—H5C	109.5	Zr1—N2—H2	106.8
H5B—C5—H5C	109.5	C5—N3—C6	110.95 (14)
N3—C5—H5A	109.5	C5—N3—Zr1	117.87 (12)
N3—C5—H5B	109.5	C6—N3—Zr1	131.02 (12)
N3—C5—H5C	109.5	C7—N4—Zr1	112.94 (10)
H6A—C6—H6B	109.5	C8—N4—C7	111.04 (14)
H6A—C6—H6C	109.5	C8—N4—Zr1	135.64 (12)
H6B—C6—H6C	109.5	C9—N5—Zr1	113.45 (11)
N3—C6—H6A	109.5	C10—N5—C9	111.11 (14)
N3—C6—H6B	109.5	C10—N5—Zr1	135.34 (11)
N3—C6—H6C	109.5	N2—Zr1—N1	172.83 (5)
H7A—C7—H7B	109.5	N3—Zr1—N1	94.35 (5)
H7A—C7—H7C	109.5	N3—Zr1—N2	92.81 (5)
H7B—C7—H7C	109.5	N3—Zr1—N4	120.99 (6)
N4—C7—H7A	109.5	N3—Zr1—N5	116.76 (6)
N4—C7—H7B	109.5	N4—Zr1—N1	88.97 (5)
N4—C7—H7C	109.5	N4—Zr1—N2	87.62 (5)
H8A—C8—H8B	109.5	N5—Zr1—N1	89.88 (5)
H8A—C8—H8C	109.5	N5—Zr1—N2	86.60 (5)
H8B—C8—H8C	109.5	N5—Zr1—N4	122.15 (6)
N4—C8—H8A	109.5		

### Hydrogen-bond geometry (Å, º)

**Table:**

*D*—H···*A*	*D*—H	H···*A*	*D*···*A*	*D*—H···*A*
N1—H1···I1^i^	1.00	2.78	3.5922 (14)	138
N2—H2···I1	1.00	2.69	3.6153 (15)	138

Symmetry code: (i) *x*−1/2, −*y*+3/2, −*z*+1.
